# Da-Bu-Yin-Wan and Qian-Zheng-San to Neuroprotect the Mouse Model of Parkinson's Disease

**DOI:** 10.1155/2014/729195

**Published:** 2014-12-24

**Authors:** Xiao-Gang Gong, Hong-Mei Sun, Yi Zhang, Shu-Jing Zhang, Yu-Shan Gao, Jing Feng, Jing-Hong Hu, Cong Gai, Zhen-Yu Guo, Hong Xu, Ling Ma

**Affiliations:** ^1^Department of Anatomy, School of Preclinical Medicine, Beijing University of Chinese Medicine, No. 11 N. 3rd Ring Eastern Road, Beijing 100029, China; ^2^Center of Scientific Research, School of Preclinical Medicine, Beijing University of Chinese Medicine, No. 11 N. 3rd Ring Eastern Road, Beijing 100029, China

## Abstract

Da-Bu-Yin-Wan (DBYW) and Qian-Zheng-San (QZS), two classic traditional Chinese medicinal formulas, were clinically employed to treat Parkinson's disease (PD). Our previous studies demonstrated neuroprotective effects of them on mitochondrial function in PD mice induced by *1-methyl-4-phenyl-1,2,3,6-tetrahydropyridine* (MPTP). The purpose of this research was to investigate their possible mechanisms in the light of mitochondrial ATP-sensitive potassium (mitoK_ATP_) channels. The neuroprotective effect of DBYW and QZS on dopamine (DA) neurons in substantia nigra (SN) in the MPTP-induced PD mice was investigated by behavioral test (pole test) and immunohistochemistry. Adenosine triphosphate (ATP) level in the midbrain tissue was detected by firefly luciferase method. MitoK_ATP_ channel subunits SUR1 and Kir6.2 mRNA and protein expressions were tested by real-time PCR (RT-PCR) and Western blot. It was observed that DBYW and/or QZS served to ameliorate MPTP-induced behavioral impairment and prevent the loss of substantia nigra dopamine neurons, as well as increase ATP level in the midbrain tissue and downregulate SUR1 expression at mRNA and protein levels with no marked influence on Kir6.2. We concluded that DBYW and QZS exhibit neuroprotective effects probably through the regulation of ATP level and mitoK_ATP_ channel subunit expressions.

## 1. Introduction

Parkinson's disease (PD) is a chronic progressive neurodegenerative movement disorder ranked as the second most common degenerative neurological disease, affecting more than 6 million people worldwide [1]. The neuropathological hallmarks are characterized by a profound and selective loss of dopaminergic neurons in the substantia nigra pars compacta (SNc) with presence of Lewy bodies and dystrophic Lewy neuritis in surviving neurons [2]. Clinical manifestations of PD include motor impairments involving resting tremor, bradykinesia, postural instability, and rigidity along with nonmotoric symptoms like autonomic, cognitive, and psychiatric problems [3]. Although different pathogenic causes have been discovered, in the majority of cases, the critical mechanisms of PD remain unknown.

ATP-sensitive potassium (K_ATP_) channels have been identified in a variety of tissues. K_ATP_ channels present a complex octameric structure consisting of four pore-forming, inwardly rectifying K^+^ channel subunits (Kir6.*x*) and four sulfonylurea receptor subunits (SUR*x*) that are members of the ATP-binding cassette superfamily. Several isoforms are identified, both for the pore-forming subunit (Kir6.1 and Kir6.2) and for the regulatory subunit (SUR1, SUR2A, and SUR2B). Two distinct K_ATP_ channels have been described in cells: membrane K_ATP_ channel and mitochondrial K_ATP_ channel [4, 5]. K_ATP_ channels were originally discovered in the heart, although particularly abundant in the central nervous system (CNS), and reach their highest levels in the SN and striatum [6, 7]. Evidence has shown that K_ATP_ channels comprising Kir6.2 and SUR1 are abundantly expressed in the nigral dopaminergic neurons [8–10]. K_ATP_ channels are considered as a potential downstream target of mitochondrial complex I inhibition [11]. In the mouse model of PD induced by MPTP, rapid ATP loss even ATP depletion, which has been observed for mitochondrial dysfunction, may contribute to further metabolic disorders and induce the unusual activated opening of K_ATP_ channels [12, 13].

Studies have provided evidence for the presence of mitoK_ATP_ channels in the inner mitochondrial membrane of various cell types. The channel has been described in liver [14], heart [15], brain [16, 17], skeletal muscle [18], and kidney [19]. It is found that brain mitochondria contain seven times more mitoK_ATP_ channels per milligram of mitochondrial protein than liver or heart [16, 20]. MitoK_ATP_ channels play the roles in controlling the mitochondrial volume, regulating the translation of metabolic status of cells, and responsing open/close channels to injury for neurodegeneration [21]. The channels are activated by a decreased ATP/ADP ratio in hypoxic/ischemic conditions. Its activation may shorten the action potential duration and reduce cellular calcium overload [22]. A recent study suggests that mitoK_ATP_ channels are also involved in DA cell death and particularly in the loss of DA neurons induced by low doses of 6-OHDA [23]. In DA neurons, mitoK_ATP_ channels directly couple the metabolic state of DA neuron to its electrical activity, and the oxidative stress sensitivity varies from K_ATP_ subunit to subunit. SUR1/Kir6.2 is considered more sensitive than the other K_ATP_ channel types [24]. These findings suggest that mitoK_ATP_ channels are related to DA neurons and abnormal mitoK_ATP_ channels may play a pathogenically important role in PD.

Da-Bu-Yin-Wan (DBYW, Great Yin Tonic Pill) and Qian-Zheng-San (QZS, Symmetry Leading Powder), two classic formulas of traditional Chinese medicine (TCM), have long been used to treat PD [25, 26]. The specific information of the medicines has been already reported [27]. Previously we demonstrated neuroprotective effects of DBYW and QZS on mitochondrial function in mouse model of PD induced by MPTP as the following: DBYW and QZS decreased the mtDNA damage, significantly improved complex I activity, regulated complexes II, III and IV to varying degrees, and synergistically upregulated the mRNA expression of ND1 [27–29]. In the present research, we further investigated effects of DBYW and QZS on midbrain ATP level and mitoK_ATP_ channel subunits SUR1/Kir6.2 in mRNA and protein levels. This was to investigate their possible mechanisms in the light of mitoK_ATP_ channels. Furthermore, additional data can be provided for considering traditional Chinese medicine as a potential regulator for mitoK_ATP_ channels in the treatment of Parkinson's disease.

## 2. Materials and Methods 

### 2.1. Chemicals

*1-Methyl-4-phenyl-1,2,3,6-tetrahydropyridine *(MPTP), paraformaldehyde (PFA), pepsin, and glycerin were purchased from Sigma-Aldrich (St. Louis, MO, USA). Analytical grade potassium chloride and sodium dihydrogen orthophosphate were purchased from Alfa Aesar (Ward Hill, MA, USA). Phosphate-buffered saline (PBS), sodium chloride, ethylene glycol, and 3,3-diaminobenzidine (DAB) were purchased from ZSGB-BIO Co. (Beijing, China). ATP assay kit was purchased from Beyotime Institute of Biotechnology (Haimen, China). Bicinchoninic acid (BCA) protein assay kit was purchased from Applygen Technologies Inc. (Beijing, China). All other reagents used in the research were obtained from Beijing Chemical Factory (Beijing, China).

### 2.2. Decoctions Preparation

The composition of DBYW and QZS was as follows (with voucher numbers): for DBYW,* Phellodendron chinense Schneid* (DBYW01-120306) 60 g,* Anemarrhena asphodeloides Bunge* (DBYW02-120306) 60 g,* Radix Rehmanniae Preparata* (DBYW03-120306) 90 g, and* Carapax Testudinis* (DBYW04-120306) 90 g; for QZS,* Rhizoma Typhonii Gigantei* (QZS01-120306) 60 g,* Bombyx Batryticatus *(QZS02-120306) 60 g, and* Scorpio* (QZS03-120306) 60 g.

All traditional Chinese medicines were purchased from Tong-Ren-Tang Drugstore (Beijing, China) and identified and authenticated by experts in pharmacognosy. The voucher specimens were deposited in our department. In preparing the decoctions, extract amounts of component herbs were weighed according to the classic percentage and mixed well. The mixtures were immersed for 30 min with 8 times volume of distilled water and then decocted twice at 100°C for 1 hour (h); this preparation method was established according to the ancient Chinese method as described previously [27]. Finally, the decoction was concentrated to 7.74 g/mL for DBYW and 3.51 g/mL for QZS. The concentration was expressed in total dry weight of the crude herbs per milliliter in decoction.

### 2.3. Animals and Treatments

Male C57BL/6 mice (8 weeks old, 20 ± 1 g) were provided from Vital River Co. (Beijing, China). Animals were habituated for 1 week to the animal colony and had free access to food and water. Animals were housed under standard conditions (temperature 21 ± 1°C, 12 h–12 h light-dark cycle). Animal experimental procedures and welfare were conducted strictly in conformity with the National Institution of Health Guide for the Care and Use of Laboratory Animals (NIH Publication number 85-23, revised 1985) and the related ethics regulations of Beijing University of Chinese Medicine. Every effort was made to minimize the number of animals used and reduce animal suffering in the study.

160 mice were randomly divided into 5 equal groups: saline-injected group (control group), MPTP-injected group (model group), MPTP-injected plus DBYW-treated group (DBYW group), MPTP-injected plus QZS-treated group (QZS group), and MPTP-injected plus combined decoction (CD, DBYW + QZS-) treated group (CD group). All groups except control group were injected intraperitoneally with MPTP at the dosage of 20 mg/kg per day for 5 consecutive days before decoction treatment [30, 31]. According to the standard dosages of the two formulas for human, we converted the standard dose of the two formulas for human into equivalent dose for mouse based on body weight [32]. Then mice of various decoction-treated groups were treated with gastric gavage of DBYW (7.74 g/kg), QZS (3.51 g/kg), and CD (DBYW, 3.87 g/kg; QZS, 1.76 g/kg), on a daily basis, for 23 days after 5-day MPTP injection, respectively. For the control and model groups, an equal volume of distilled water was administered by gastric gavage instead of decoction.

### 2.4. Behavioral Test

The behavior was evaluated with the pole test. The method was performed as described previously [33] with minor modifications. The mouse was placed head-downward on the top of a vertical rough-surfaced pole (diameter 2.5 cm; height 60 cm), which was wrapped in gauze to prevent slipping and the time it took to reach the floor was determined. Every animal was subjected to three trials at 3-minute intervals, and the average time was recorded. Meanwhile, their ability shown during the climbing was evaluated from the perspective of behavior. The specific scoring standard is as follows: with frequent tremors and limb stiffness, the mice lose the ability to move or die (Grade I, scores 2.5); with frequent tremors and limb stiffness, the mice were unable to hold the pole, and they fall from the top immediately (Grade II, scores 2); the mice fall before reaching the bottom after sliding and tremors (Grade III, scores 1.5); the mice climb from top to bottom, interrupted with several pauses in a spiral downward style with occasional posterior-limb slide (Grade IV, scores 1); the mice climb downward step by step in a spiral way with the presence of ability to slide with posterior limbs (Grade V, scores 0.5); the mice use four limbs, climbing from top to bottom within one effort (Grade VI, scores 0) [34]. Mice were habituated to the task in 2 trials per day for 2 days before saline or MPTP injections. The pole test for first time was performed before first saline or MPTP injection. The test for the second time was performed 6 h after the first saline or MPTP injection. The rest of pole tests were performed once every two days until mice were euthanized. All pole tests were carried out between 9 am and 12 am for each group.

### 2.5. Immunohistochemistry Analysis

To assess the effects of decoctions on the expression of tyrosine hydroxylase (TH), a marker of dopaminergic neurons, immunohistochemistry was carried out as described previously [35, 36]. After the mice were deeply anesthetized with an intraperitoneal injection of chloral hydrate (0.4 g/kg), they received a thoracotomy and were perfused through the left ventricle, first with 100 mL of saline and then with 100 mL 4% paraformaldehyde in PBS (0.01 M, pH 7.4). Then the brains were removed immediately and postfixed in 4% paraformaldehyde for 12 h. After dehydrating through an ascending series of ethanol solutions and embedding in paraffin, coronal sections were taken 1.5 mm behind optic chiasma. Sections (6 *μ*m thick) were treated with a 1% solution of H_2_O_2_ in methanol for 10 min to quench endogenous peroxidase. These slices were digested with pepsin (10 min, 25°C) and blocked with 10% normal goat serum (30 min, 37°C), followed by an overnight incubation with a mouse monoclonal antibody against TH-16 (1:400; Sigma-Aldrich, St. Louis, MO, USA) at 4°C. After rinsing with PBS, sections were incubated with biotin labeling goat anti-mouse IgG (1:200; ZSGB-BIO, Beijing, China). Then horseradish peroxidase-linked streptavidin (ZSGB-BIO, Beijing, China) was added. After the reaction, diaminobenzidine (ZSGB-BIO, Beijing, China) was used as chromogen. Finally, the numbers of positive neurons of TH were determined randomly in six sights under 10x objective lens of each section for quantitative analysis. Neuron counting was analyzed by the Image-Pro Plus, version 6.0 (Media Cybernetics, Bethesda, MD, USA).

### 2.6. ATP Level Determination

ATP level was measured by a firefly luciferase based ATP assay kit according to the manufacturer's instructions. After rinsing with PBS, each 20 mg brain tissue was mixed with 100 *μ*L lysate. After lysis, the mixtures were centrifuged at 12,000 g for 10 min at 4°C while the ATP detection working solution was incubated in white 96-well plates for 5 minutes to reduce the background of ATP. The supernatant of the samples was then transferred into white 96-well plates for 2 s and relative luminescence units (RLU) were immediately measured using a Tecan Safire 2 multifunctional microtiter plate reader (Tecan, Männedorf, Switzerland). Standard curves for the quantification were also generated using known amounts of an ATP standard and the protein concentration of each treatment group was determined using the BCA protein assay kit. Total ATP levels were expressed as relative RLU/mg protein (*μ* mol/mg protein).

### 2.7. Analysis by Quantitative Real-Time PCR

Total RNA from midbrain of mice was isolated by a standard procedure using TRIzol reagent (Invitrogen, Carlsbad, CA, USA). First-strand cDNA was synthesized by a 20 *μ*L reaction mixture containing 1 *μ*g of total RNA using the moloney murine leukemia virus reverse transcriptase system (Invitrogen, Carlsbad, CA, USA) according to the manufacturer's protocol. Oligonucleotide primers for SUR1 and Kir6.2 were selected using the DNA sequences in Mouse Genome Database of PubMed (MGI ID: 1352630 and MGI ID: 107501, resp.). Sequences of the real-time PCR specific primers were 5′-CGG GTT CAC ACC ATT CTG AC′ (forward) and 5′-CGA GGC AAA CAC TCC ATC TT-3′ (reverse) for SUR1 and 5′-GGC CAG GAA AGC TAC TTA GAC-3′ (forward) and 5′-GGC CAG ACA GAC AGA GAA TG-3′ (reverse) for Kir6.2. The real-time PCR reaction was carried out under the conditions recommended by the manufacturer (Life Technologies, Inc.) A 25 *μ*L reaction mixture containing forward and reverse primers (0.15 *μ*M each), 1 *μ*L of cDNA (10x diluted), and 2x SYBR Green PCR master mix (Toyobo, Osaka, Japan) was amplified under the following conditions: initial denaturation at 95°C for 5 min followed by 40 cycles of 15 s at 95.0°C, 35 s at 59.5°C, and 30 s at 72.0°C in ABI 7500 sequence detection system (Applied Biosystems, Foster City, CA, USA) according to the manufacturer's instructions. Each cDNA sample assayed for SUR1/Kir6.2 mRNA was also subjected to *β*-actin analysis. All amplification reactions were performed in triplicate to achieve reproducibility. The quantification data were analyzed with Sequence Detection software, version 1.3 (Applied Biosystems, Foster City, CA, USA). To confirm the amplification specificity, a melting curve analysis was added after thermocycling for every reaction, determining dissociation of the PCR amplified products from 60°C to 95°C. The negative controls lacking template cDNA were included in each experiment. The mean value of the replicates for each sample was calculated and expressed as the threshold cycle (*C*_*T*_). The fold change in the level of SUR1/Kir6.2 between MPTP-treated group and control group, normalized by the level of *β*-actin, was determined by the 2^−ΔΔ*C*_*T*_^ method [37], where Δ*C*_*T*_ = *C*_*T*,SUR1/Kir6.2_ − *C*_*T*,*β*-actin_ and ΔΔ*C*_*T*_ = Δ*C*_*T*,MPTP treatment_ − Δ*C*_*T*,control_.

### 2.8. Analysis of Protein Expression of SUR1 and Kir6.2 by Western Blot

To study the effects of decoctions on the expression of mitoK_ATP_ subunits SUR1/Kir6.2 in the mice model of PD, mitochondria were isolated from midbrain tissue and protein was extracted with the mitochondrial protein extraction kit (Genmed, Arlington, MA, USA) according to standard protocols. The protein content of all samples was determined using the BCA protein assay (Applygen Technologies Inc., Beijing, China), and equal amounts of protein (30 mg) were separated on a 10% sodium dodecyl sulfate polyacrylamide gel electrophoresis (SDS-PAGE). Proteins were then electrotransferred onto a polyvinylidene difluoride membrane (Millipore, Bedford, MA, USA) and blocked with 5% nonfat milk in Tris-buffered saline-Tween 20 (TBS-T) for 1 h at room temperature. Membranes were then incubated with primary antibodies: anti-SUR1 (catalog #ab32844, 1 : 750 dilution; Abcam, Cambridge, UK), anti-Kir6.2 (catalog #ab79171, 1 : 750 dilution; Abcam, Cambridge, UK), and anti-*β*-actin (1 : 2000 dilution; ZSGB-BIO, Beijing, China) overnight at 4°C in TBS-T and 0.5% nonfat milk. This was followed by incubation with corresponding horseradish peroxidase-coupled secondary antibodies (1 : 2000 for 1 h at room temperature; ZSGB-BIO). The enhanced chemiluminescent (ECL) plus detection reagent (Applygen Technologies Inc., Beijing, China) was applied on the membrane, and the chemiluminescence was visualized by a Tanon-4500 digital image processing system (Tanon, Shanghai, China). Data were analyzed and quantified using Quantity One 4.6.2 application software (Bio-Rad, Hercules, CA, USA).

### 2.9. Statistical Analysis

All results were expressed as mean ± SEM (standard error of the mean). Statistically significant differences between groups were analyzed by one-way analysis of variance (ANOVA), with Dunnett's *t*-test. For real-time PCR results, a two-tailed independent-samples *t*-test was used for statistical analysis of the comparative data from two groups. All data were processed with GraphPad Prism version 5.0 software (GraphPad, San Diego, CA, USA). A difference of *P* < 0.05 was considered to be statistically significant.

## 3. Results

### 3.1. Behavioral Test

**Figure 1:**
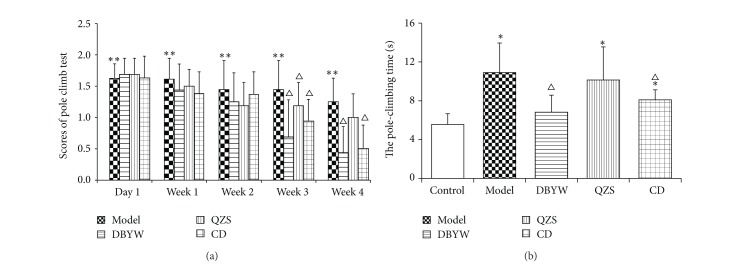
Behavioral test. (a) The scores of pole climb test and (b) the time to complete the pole test. Results are presented as mean ± SEM (*N* = 8, ^*^ *P* < 0.05 versus control group, ^△^ *P* < 0.05 versus model group). DBYW: Da-Bu-Yin-Wan; QZS: Qian-Zheng-San; CD: combined decoction.

MPTP injections resulted in significant motor deficits as evaluated by the pole test. Pole test performance is presented in Figure 1. Before and after giving normal saline, mice from control showed no difference in the results from pole climb tests. At the very day of intraperitoneal injection of MPTP and the following several days, they showed obvious decrease in ability of coordination, manifesting as fast sliding, lack of ability to hold the pole, and falling. The model group gained more scores during 1 to 4 weeks than the control group (*P* < 0.05). Mice from each treatment group scored lower than the model group with no statistical significance observed in the first week. The scores gained by DBYW group during 2 to 4 weeks were notably lower than the model group with a trend for decreasing. The scores of QZS group showed a trend for decreasing in comparison with model group, while no statistical significance can be observed. As such, each treatment group began to recover in the 1st week with most obvious period of recovery being observed during 3 to 4 weeks. The scores gained by groups were compared on the first day (6 h after intraperitoneal injection of MPTP) and the first week to the fourth week; results are shown in Figure 1(a). The pole-climbing time was increased by 96.6% in MPTP-treated mice, compared with untreated controls (*P* < 0.05). In addition, the climbing time was reduced by 37.5% and 25.9% in mice treated with DBYW and CD (*P* < 0.05), compared with MPTP-treated mice. The effect of QZS achieved no statistical significance (Figure 1(b)).

### 3.2. Analysis of the Expression of TH in the SN by Immunohistochemistry

**Figure 2:**
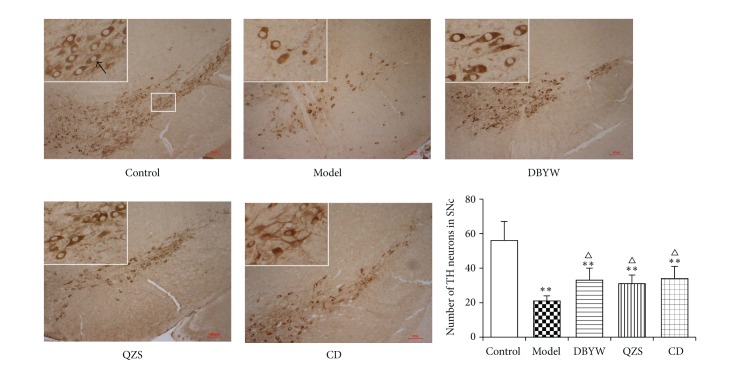
Immunohistochemistry analysis revealed differential number of TH immunopositive neurons in the SN of various treatment groups. Area within rectangle is shown in higher magnification at the top left corner of each picture. The black arrow points to typical TH-positive neurons. Scale bar: 100 *μ*m. Data are expressed as mean ± SEM (*N* = 6, ^**^ *P* < 0.01 versus control group, ^△^ *P* < 0.05 versus model group). DBYW: Da-Bu-Yin-Wan; QZS: Qian-Zheng-San; CD: combined decoction.

The loss of dopaminergic neurons was assessed by immunohistochemistry analysis of TH expression in the anatomical region of SN. As shown in Figure 2, MPTP administration caused a significant loss of TH neurons compared to control group in the mice SN. However, treatment with various decoctions rescued MPTP-induced neuron loss in the SN compared to model group, respectively. The numbers of TH-positive neurons of model group were significantly decreased by 55.6% compared to control group (*P* < 0.01). The numbers were significantly increased by 36.4%, 30.3%, and 38.2% in mice treated with DBYW, QZS, and CD compared to model group, respectively (*P* < 0.05).

### 3.3. ATP Level Determination

**Figure 3:**
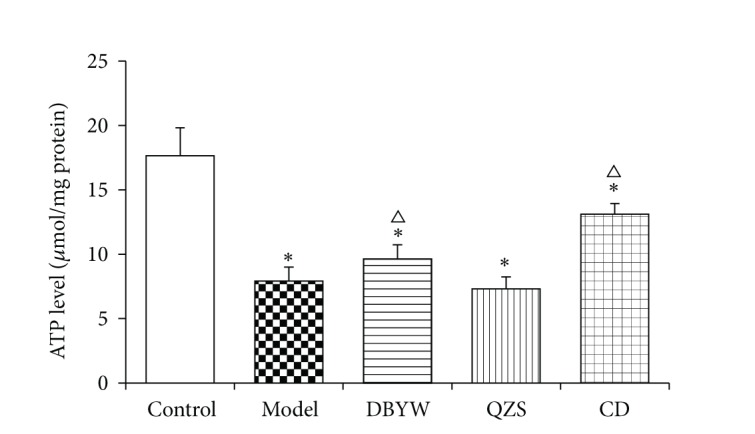
The ATP levels in mice midbrain. The mean ± SEM (*N* = 6, ^*^ *P* < 0.05 versus control group, ^△^ *P* < 0.05 versus model group), expressed as relative luminescence units (RLU)/mg protein (*μ* mol/mg protein). DBYW: Da-Bu-Yin-Wan; QZS: Qian-Zheng-San; CD: combined decoction.

As can be seen in Figure 3, the ATP levels in MPTP-treated mice midbrain were statistically lower than that of controls, reduced by 55.57% (*P* < 0.05). The ATP levels were significantly increased by 17.65% and 39.51% in mice treated with DBYW and CD (*P* < 0.05), compared to model group. No significant difference was noted between ATP levels of QZS group and model group.

### 3.4. Analysis of mRNA Expression of SUR1 and Kir6.2 by Quantitative Real-Time PCR

**Figure 4:**
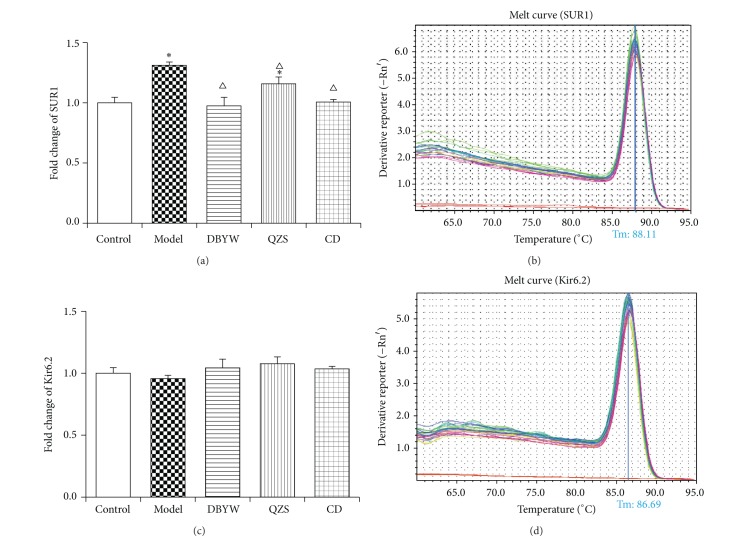
(a, c) Effects of decoctions on mRNA levels of SUR1 and Kir6.2 measured by real-time PCR. The fold changes of SUR1 and Kir6.2 expressions are calculated by the 2^−ΔΔ*C*_*T*_^ method and expressed as mean fold induction over control group that has been normalized to 1.0. Data are expressed as mean ± SEM (*N* = 5, ^*^ *P* < 0.05 versus control group, ^△^ *P* < 0.05 versus model group). DBYW: Da-Bu-Yin-Wan; QZS: Qian-Zheng-San; CD: combined decoction. (b, d) Melting curve analysis of SUR1 and Kir6.2. Tm: temperature of melting.

To investigate the effect of decoctions on SUR1/Kir6.2 expression at the mRNA level, we performed quantitative real-time PCR. As shown in Figure 4(a), MPTP increased (31.0%) mRNA level of SUR1 significantly (*P* < 0.05). Various-decoction treatments (DBYW, QZS, and CD) remarkably decreased mRNA level of SUR1 by 34.0%, 18.1%, and 29.7% compared with model group, respectively (*P* < 0.05). There were no significant intergroup differences in mRNA level expressions with respect to Kir6.2 (Figure 4(c)).

In addition, a melting curve analysis was performed which resulted in single product-specific melting temperatures: 88.1°C (SUR1), 86.7°C (Kir6.2), and 89.3°C (*β*-actin), respectively. No primer-dimer formations were generated during the applied 40 real-time PCR amplification cycles. Melting curve of SUR1/Kir6.2 is shown in Figures 4(b) and 4(d); melting curve of *β*-actin is not shown.

### 3.5. Analysis of Protein Expression of SUR1 and Kir6.2 by Western Blot

**Figure 5:**
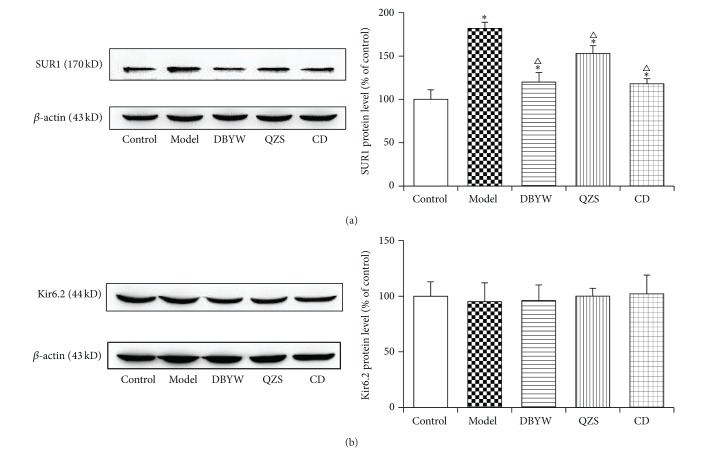
Effects of decoctions on protein levels of SUR1 (a) and Kir6.2 (b) measured by Western blot. Density values for each band were first normalized to loading control (*β*-actin) and then to the control group. Data are expressed as mean ± SEM (*N* = 5, ^*^ *P* < 0.05 versus control group, ^△^ *P* < 0.05 versus model group). DBYW: Da-Bu-Yin-Wan; QZS: Qian-Zheng-San; CD: combined decoction.

To investigate the effect of decoctions on SUR1/Kir6.2 expression at the protein level, we performed Western blot. As shown in Figure 5(a), the SUR1 protein level in the model group increased (31.0%) significantly compared with the control group (*P* < 0.05). Various-decoction treatments (DBYW, QZS, and CD) remarkably decreased protein level of SUR1 by 34.0%, 18.1%, and 29.7% compared with model group, respectively (*P* < 0.05). Moreover, an increasing trend of the protein level of SUR1 was also noted from the QZS to DBYW and to CD treatment groups (*P* < 0.05). There were no significant differences among groups in Kir6.2 protein level expression (Figure 5(b)).

## 4. Discussion

In this study, we have shown that DBYW and QZS suppressed MPTP-induced mitoK_ATP_ channel subunit SUR1 mRNA and protein overexpression in the mouse midbrain. When compared to the control group, the model group had significantly higher expressions of SUR1 mRNA and protein. Treatment with various decoctions significantly decreased SUR1 mRNA and protein levels, above those in the model group (all *P* < 0.05). However, there were no significant intergroup differences in mRNA and protein level expressions with respect to Kir6.2.

K_ATP_ channels existed in multiple parts of the cells, such as the surface of the plasmalemmal membrane and the inner mitochondrial membrane (mitoK_ATP_ channels). It is found that K_ATP_ channels are particularly abundant in the CNS and reach their highest levels in the SN and striatum [6, 7]. K_ATP_ channels that comprise SUR1 and Kir6.2 are abundantly expressed in the nigral dopaminergic neurons [11, 38]. The mitoK_ATP_ channel is thought to have similar structure with both Kir and Sur subunits, although the exact composition of the mitoK_ATP_ has not been determined [23]. Studies have shown that brain mitochondria contain far more mitoK_ATP_ channels than liver or heart, playing, presumably, an important role for mitoK_ATP_ channels in neurons [16, 20]. The tissue selective expression of the subunits leads to the predominance of different types of K_ATP_ channels which may be involved in distinct physiological processes. Therefore, we focused on mitoK_ATP_ subunits SUR1/Kir6.2 in the present study.

Mitochondria are now considered to play a crucial role in the development and progression of neurodegenerative diseases such as PD and Alzheimer's disease through initiating different signaling cascades. In PD, the dysfunction of the mitochondrial electron transport chain leads to the increase of reactive oxygen species or metabolic stress and renders subsets of selectively vulnerable neurons intrinsically susceptible to cellular degeneration and oxidative stress [39, 40]. MitoK_ATP_ channels exert important function in controlling the mitochondrial volume, regulating the translation of metabolic status of cells, and responsing open/close channels to injury for neurodegeneration [24]. MitoK_ATP_ channels were considered a potential target for neuroprotection in PD and aging. Recent studies have shown that inhibition of mitoK_ATP_ channels with 5-hydroxydecanoic acid inhibited the increase in dopaminergic cell death induced by angiotensin II, as well as the increase in superoxide/superoxide-derived reactive oxygen species levels and the angiotensin II-induced decrease in the mitochondrial inner membrane potential in cultured dopaminergic neurons [41]. Evidence has shown that, after acute rotenone-induced K_ATP_ channel activation in mouse brain slices, only highly responsive dopaminergic neurons express the K_ATP_ channel subunits SUR1 and Kir6.2 [42–44]. In DA neurons, mitoK_ATP_ directly couple the metabolic state of DA neuron to its electrical activity, and the oxidative stress sensitivity varies from K_ATP_ subunit to subunit. SUR1/Kir6.2 is considered more sensitive than the others. Research shows that short-term activation of mitoK_ATP_ can effectively antagonize the toxicity of neurotoxins on cells, but the long-term effect, just to the opposite, may promote the death of DA neurons [24]. In the present research, the result of RT-PCR is consistent with that of Western blot showing that the SUR1 mRNA and protein level were markedly increased in the midbrain of MPTP-treated mice compared with control group (*P* < 0.05). SUR1 expression was significantly decreased in DBYW, QZS, and CD groups compared with the model group (*P* < 0.05). Our research demonstrated that mitoK_ATP_ channel expressed SUR1/Kir6.2 subunit in mice brain. Moreover, DBYW and QZS downregulated MPTP-induced SUR1 mRNA and protein overexpression in the PD mice midbrain. As has been proved, mitoK_ATP_ channel is formed from two dissimilar subunits—Kir6.*x* subunits that generate the morphological structure of channels and SUR*x* subunits that generate the regulatory role. Specifically, there is evidence that SUR1 serves as the regulatory subunit of the K_ATP_ channels in some types of neurons [45]. And this is probably the reason why in the present study Kir6.2 expressions did not show marked intergroup differences. We deduce that SUR1, rather than Kir6.2, mediates the regulatory roles of MPTP and DBYW/QZS on mitoK_ATP_.

In mouse PD model induced by MPTP, rapid ATP loss even ATP depletion which has been observed for mitochondrial dysfunction may contribute to further metabolic disorders and induce the unusual activated opening of K_ATP_ channels [12, 13]. The results of the present study demonstrated that the ATP level was decreased evidently in MPTP-induced mice model compared with controls (*P* < 0.05). ATP levels of various decoctions groups were significantly increased compared to model group (*P* < 0.05). MPTP, an inhibitor of mitochondrial respiratory chain complex I, is a potent neurotoxin extensively used to induce a Parkinsonian animal model [46, 47]. Complex I derangement causes impairment of mitochondrial function and defects of energy metabolism that finally lead to neuronal death [8, 48]. Therefore, it suggests that MPTP induced the decrease of ATP production, leading to continuous unexpected activation of mitoK_ATP_ channel that further consumes ATP and then to the deterioration of mitochondrial dysfunction, followed by the death of DA neurons. The mechanism of mitoK_ATP_ activation may be selective upregulation of mitoK_ATP_ subunits of SUR1; DBYW and QZS, through antagonizing the upregulation of SUR1, regulate the mitoK_ATP_ channel to achieve the function of DA neuron protection. As a marker protein of dopaminergic neurons, tyrosine hydroxylase (TH) is decreased in the nigrostriatal DA neurons of PD patients [49] and abnormal expression of TH in the SN suggested that injury factors caused irreversible damage to dopaminergic neurons [50]. Therefore, correlated with PD pathogenesis, the nigrostriatal neurodegenerative changes were inferred from the number of TH cells in the SN and the DA tissue levels in the striatum. In this study, the numbers of TH-positive neurons were counted and the results are consistent with our previous study [27] that DBYW, QZS, and CD prevented the MPTP-induced loss of TH-positive neurons in SN (*P* < 0.05; Figure 2).

The pole test, a method to evaluate the behavior in animals proposed by Ogawa et al. in 1985, is an extensively used method for evaluating the mouse movement disorder caused by striatal dopamine depletion [51]. In the present study, we prioritized the assessment by simultaneously recording the pole climb time and behavioral score. Because of the individual difference and the pole sliding as a result of injury from modeling, the result of pole climb may be not thoroughly exact. Therefore, we combined the results from pole climb duration and the scores of pole climb of the mice, so that more exact results can be achieved. The results show that DBYW and CD significantly improved the behavioral impairment induced by MPTP (*P* < 0.05). The effect of QZS on behavioral test did not achieve statistical significance.

Traditional Chinese medicine is extensively employed and especially valued for thousands of years and proved to have fewer side effects than chemical medicines to a certain extent [52]. In the present research, our focus was on DBYW and QZS, two classic TCM formulas.* Anemarrhena asphodeloides Bge*, an ingredient in DBYW, has been demonstrated to promote the survival of dopaminergic neurons by the action of its compound—*Mangiferin* [53, 54]. Treated by DBYW combined with Madopar, parkinsonism in PD patients was improved significantly compared to controls [55]. It is reported that Rhizoma Typhonii Gigantei, as one of the ingredients of QZS, functions to stimulate the mouse spleen cells and human lymphocytes and hence regulate immunological function [56].* Scorpio*, another component of QZS, contains a kind of polypeptide toxin, scorpion venom component III, which protects dopaminergic neurons of SN compact region of midbrain and ameliorates the locomotor disability and spatial learning memory deficits induced by MPTP [57]. In our research, it has been proven that DBYW and QZS are neuroprotective to varying extent. Specifically, DBYW functions advantageously as compared to QZS. This may be because the former contains more neuroprotective ingredients such as* Mangiferin* than the latter based on pharmaceutical research referred to above. Mice from CD group in comparison to those from DBYW or QZS group were observed with favorable behavioral manifestations, protection for DA neurons, and increase in ATP levels; on the other hand, they, in comparison to DBYW group, exhibited insignificant differences with respect to the regulation of SUR1/Kir6.2 expressions. This suggests some concomitant effect (not simple synergistic effect) when the two are used in combination, although we have noticed that the combined usage of the prescriptions does not equal a simple efficacy sum-up, which means their combination may be involved in various mechanisms.

In the present research, for the first time, the possible mechanism of DBYW and QZS to possess neuroprotective properties is demonstrated from the perspective of mitoK_ATP_ channel. In addition, further researches are in progress to elucidate the isolation and effects of chemical constituents of DBYW or QZS.

## 5. Conclusions

In summary, the present study demonstrated that DBYW and/or QZS served to ameliorate MPTP-induced behavioral impairment, prevent the loss of substantia nigra dopamine neurons, increased ATP level in the midbrain tissue of PD mice, and downregulated SUR1 expression at mRNA and protein levels. We conclude that both DBYW and QZS exhibit neuroprotective effects with DBYW exhibiting significantly better efficacy in most parameters, and the combined decoction appears to be more promising as DA neurons protection compared with individual DBYW or QZS, and that this process is likely to be mediated by mitoK_ATP_ SUR1 rather than Kir6.2.

## Abbreviations

ATP: Adenosine triphosphate
CD: Combined decoction
CNS: Central nervous system
DA: Dopamine
DBYW: Da-Bu-Yin-Wan
MitoK_ATP_: Mitochondrial ATP-sensitive potassium
MPTP: 1-Methyl-4-phenyl-1,2,3,6-tetrahydropyridine
One-way ANOVA: One-way analysis of variance
PBS: Phosphate-buffered saline
PD: Parkinson’s disease
QZS: Qian-Zheng-San
RLU: Relative luminescence units
SEM: Standard error of the mean
SNc: Substantia nigra pars compacta
TCM: Traditional Chinese medicine
TH: Tyrosine hydroxylase.
